# A Burst-Based “Hebbian” Learning Rule at Retinogeniculate Synapses Links Retinal Waves to Activity-Dependent Refinement 

**DOI:** 10.1371/journal.pbio.0050061

**Published:** 2007-03-06

**Authors:** Daniel A Butts, Patrick O Kanold, Carla J Shatz

**Affiliations:** Department of Neurobiology, Harvard Medical School, Boston, Massachusetts, United States of America; Salk Institute for Biological Studies, United States of America

## Abstract

Patterned spontaneous activity in the developing retina is necessary to drive synaptic refinement in the lateral geniculate nucleus (LGN). Using perforated patch recordings from neurons in LGN slices during the period of eye segregation, we examine how such burst-based activity can instruct this refinement. Retinogeniculate synapses have a novel learning rule that depends on the latencies between pre- and postsynaptic bursts on the order of one second: coincident bursts produce long-lasting synaptic enhancement, whereas non-overlapping bursts produce mild synaptic weakening. It is consistent with “Hebbian” development thought to exist at this synapse, and we demonstrate computationally that such a rule can robustly use retinal waves to drive eye segregation and retinotopic refinement. Thus, by measuring plasticity induced by natural activity patterns, synaptic learning rules can be linked directly to their larger role in instructing the patterning of neural connectivity.

## Author Summary

The brain is comprised of an immense number of connections between neurons, and clever strategies are required to achieve the correct wiring during development. One common strategy uses neural activity itself as feedback to instruct individual connections (synapses) through synaptic learning rules that delineate which patterns of activity strengthen the synapse and which weaken it. Throughout life, such activity-dependent synaptic changes are likely driven by experience and are thought to underlie learning and memory, but during early stages of development, they are often driven by activity spontaneously generated within the brain. Here, we study connections in the visual pathway between the retina and lateral geniculate nucleus (LGN), which—to develop correctly—require spontaneous “retinal waves” before the eye is responsive to light. By replaying the retinal wave activity as it appears at single LGN synapses, we observe a novel learning rule that describes a relatively simple computation for the developing synapse in the context of retinal wave activity. We then demonstrate how this learning rule is matched to properties of the retinal waves in order to robustly drive the synaptic refinement that occurs in the visual system.

## Abstract 3

A novel learning rule describes a simple computation by which retinal wave activity robustly drives the synaptic refinement that occurs in the visual system.

## Introduction

Though synaptic plasticity is a feature of most excitatory synapses in the brain, how it functions in realistic contexts is largely unclear because its effects usually only manifest on the system level. The synaptic refinement of retinal ganglion cell (RGC) axons in the developing lateral geniculate nucleus (LGN) during early postnatal development in rodents provides an opportunity to study synaptic plasticity in this larger context, since the activity over the inputs to the LGN and the resulting developmental outcome are both well characterized [1]. At the earliest ages studied, LGN neurons receive inputs from a large number of RGCs from both eyes, and this number reduces to one or a few over the course of development [2–5]. At the system level, this synaptic refinement results in segregation into eye-specific regions and establishment of fine retinotopy, with neighboring RGCs projecting to neighboring LGN neurons [6].

This synaptic refinement—as well as similar refinement in the developing visual cortex [7,8] and superior colliculus [9]—is known to require spontaneously generated activity in the developing retina [10,11]. This activity consists of correlated bursts of action potentials that spread across large regions of the retinal ganglion cell layer [12]. These retinal waves have distinct spatiotemporal properties that have been studied in detail through a variety of multi-electrode [13,14] and calcium imaging studies [12,15,16].

Some aspects of the retinal wave activity, such as coincidences of RGC activity over second-long time scales, specifically contain information that could instruct synaptic refinement [14,17]. However, it is not known whether developing retinogeniculate synapses actually use the information available from retinal wave activity [18]. At one extreme, retinal activity may just be permissive, such that it is required for RGC axons to recognize chemical markers or stimulate outgrowth, but provides no additional instructions for development [18,19]. However, several recent studies have manipulated retinal wave activity and shown that some aspects of its spatiotemporal patterning are necessary for synaptic refinement [20,21], suggesting an instructive role for retinal waves. It is generally thought that such instruction manifests at individual synapses as a synaptic learning rule that translates specific patterns of pre- and postsynaptic activity that arise from retinal waves into long-lasting changes in synaptic strength, ultimately resulting in stabilization of correctly projecting synapses and elimination of incorrect connections [22]. Although many different forms of synaptic learning rules have been observed at synapses throughout the brain [23]—including at the retinogeniculate synapse [3,24]—it is unclear how any such rule would operate in the context of the complex spatiotemporal patterning of activity provided by retinal waves.

Here, we report a novel learning rule measured at the retinogeniculate synapse that is based on the system-level patterning of neuronal activity generated by retinal waves in vivo. This learning rule demonstrates different amounts of long-term synaptic strengthening and weakening based on the timing of bursts over a seconds-long temporal window. It thus is qualitatively different from previously observed forms of synaptic plasticity, though we demonstrate how it is consistent with spike-time dependent plasticity [25]. Importantly, because burst-time–dependent plasticity (BTDP) describes the changes that synapses make in response to realistic activity, we demonstrate that it leads to the developmental refinement of the LGN observed on the system level and, furthermore, suggests how to predict the results of such alterations in activity [7,9,19–21,26,27]. In doing so, we present a full description of how natural activity patterns likely guide system-wide retinogeniculate refinement through changes in synaptic efficacy at the synapse level.

## Results

### Measuring Synaptic Plasticity in the Context of Realistic Activity Patterns

**Figure 1:**
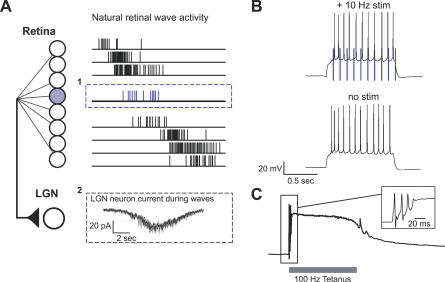
Generating In Vivo Activity Patterns (A) Schematic demonstrating natural activity resulting from retinal waves in the retina (top) and LGN (bottom): a retinal wave involves activity over a population of RGCs (#1) that evokes a large synaptic input in target LGN neurons (#2). Dashed boxes correspond to the two components of natural retinal wave activity reproduced in our experiments. Retinal wave multi-electrode recording data were adapted from Wong et al. [14]; LGN synaptic recording adapted from Mooney et al. [30] (B) Retinal wave activity at the retinogeniculate synapse is reproduced by minimal 10-Hz stimulation to the OT (vertical blue lines) paired at a given latency with direct current injection into the recorded LGN neuron to evoke 10–20 Hz bursting (top). Participation of the selected synapse has negligible effect on LGN firing, as shown by comparing the depolarization paired with +100 ms latency OT stimulation (stim) (top) with depolarization alone (bottom). (C) This situation is in marked contrast to a tetanus protocol, which involves higher current stimulation (100 Hz for 1 s), resulting in a long-lasting depolarization largely absent of postsynaptic spiking. Scale bars for (B) and (C) are shown between these panels.

Using perforated patch recording from an in vitro slice preparation of the LGN and optic tract (OT), the effects of the natural activity patterns produced by retinal waves on selected retinogeniculate synapses were examined. Throughout most of the period of eye segregation, RGCs provide the major source of driven input to the LGN [28,29]; as a result, the population-level imaging of retinal waves provides a full view of the spatiotemporal dynamics across the inputs to LGN neurons (Figure 1A). In LGN neurons, these inputs manifest as large synaptic currents lasting seconds, and evoke bursts of action potentials in response [30]. Thus, to replicate the effects of retinal waves on the synapse, we combined minimal stimulation of the OT (to activate one or a few synapses) with direct current injection into the LGN neuron (to simulate the remainder of the inputs). Such current injection was adjusted to evoke physiologically appropriate LGN activity: 10–20 Hz spiking for 1 s [30].

Though both the participation of individual RGCs in retinal waves [14] and the retinal waves themselves [16] are highly variable, previous analysis of the spatiotemporal properties of the retinal waves demonstrated that the information relevant for driving synaptic refinement is only contained in the relative timing of bursts, rather than other details of the bursts themselves [17]. Thus, to tie changes in the retinogeniculate synaptic efficacy to instructive features of the retinal wave activity, we measure how the strength of the selected synapses are affected by particular “burst latencies” between presynaptic OT stimulation (10 Hz for 1 s) and postsynaptic depolarization (Figure 1B). Stimulation of the synapses activated via OT stimulation has little effect on the firing of the postsynaptic neuron, due to the small amplitude of synaptic inputs, as shown in a comparison between the firing pattern of the neuron with (Figure 1B, top) and without (Figure 1B, bottom) OT stimulation. This is in marked contrast to that of tetanic stimulation, which dramatically alters postsynaptic activity patterns (Figure 1C).

**Figure 2:**
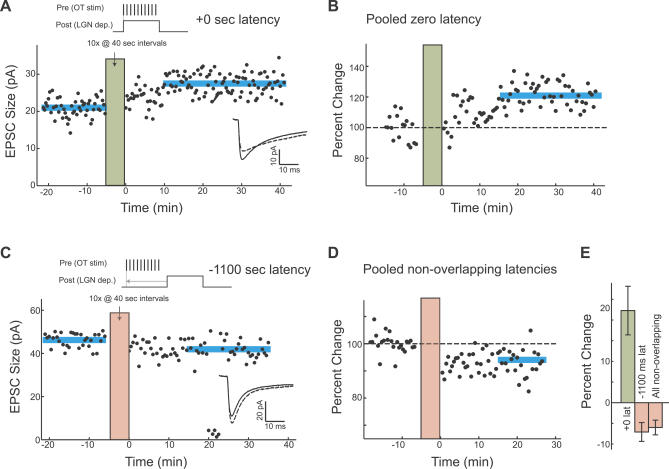
Bidirectional Synaptic Plasticity Evoked by Natural Activity Patterns (A) The maximum current during EPSCs evoked every 30 s throughout the course of a zero-latency pairing experiment (with simultaneous pre- and postsynaptic bursts). Inset: average EPSCs before (dashed) and after (solid) stimulation. As with (B–D), horizontal blue lines represent the mean EPSC size, and vertical green and red boxes demonstrate the duration of the stimulation protocol. dep., depolarization; stim, stimulation. (B) Summary of all zero-latency experiments, showing an average increase in synaptic efficacy of 21.3% (*n* = 7 neurons). (C) A single experiment showing EPSC size for a −1,100-ms latency pairing experiment. (D) Summary over all non-overlapping experiments shows an average −5.9% change in EPSC size (*n* = 13 neurons). (E) The zero-latency and non-overlapping burst protocols evoke significant changes in synaptic efficacy: summary plot with standard error is shown.

Despite the small contributions of synaptic stimulation to the firing of the postsynaptic neuron, pairing pre- and postsynaptic bursts reliably leads to long-lasting increases in the efficacy of the synapse, as shown in an example in which presynaptic stimulation and postsynaptic depolarization were completely overlapping (Figure 2A). The increase in the strength of the synapse resulting from this “zero latency” stimulation is usually gradual (over the course of 15 min) and modest (31.0% in this example). This time coarse is in marked contrast to that of long-term potentiation (LTP) induced by tetanic stimuli, which typically leads to an initial 2- to 3-fold increase in excitatory postsynaptic current (EPSC) size and then a long-lasting increase as high as 100% (as in a previous study of plasticity in the LGN [24]). In contrast, the average magnitude of synaptic enhancement that we measured was 21.3 ± 5.1% (*n* = 7), and this increase in efficacy builds gradually over roughly 15 min (Figure 2B).

This modest enhancement of synaptic efficacy was only observable using perforated patch recording, and was not seen during standard whole-cell recording or following a patch rupture during a perforated patch recording. Furthermore, as a result of the small synaptic currents observed (typically ~20 pA), small soma size (mean membrane resistance of 925 MΩ), and high serial resistance (*R*_S_ = 50–100 MΩ) typical of recordings from these very immature neurons, a large number of recordings contained changes in EPSC size that resulted from fluctuations in *R*_S_ and/or patch rupture. As a result, strict criteria were applied to eliminate more than 90% of the recordings due to these variable factors (see Materials and Methods).

To determine whether the observed synaptic enhancement depends on coincidence of pre- and postsynaptic bursts or simply on the occurrence of presynaptic stimulation and/or postsynaptic depolarization, we performed the identical stimulation protocol described above with a latency of −1,100 ms; in this case, there is no overlap between pre- and postsynaptic activity because presynaptic stimulation ends 100 ms before postsynaptic depolarization begins. This “non-overlapping” latency caused a modest decrease in synaptic efficacy (Figure 2C), which was consistent across *n* = 6 experiments performed at this latency (−6.2 ± 1.7%). Such a decrease was consistent with a range of other non-overlapping latencies used, including +1,100-ms latency (−3.5%, *n* = 3), +1,200-ms latency (−2.1%, *n* = 1), +1,500-ms latency (−10.9%, *n* = 1), −2,100-ms latency (−10.7%, *n* = 1), and presynaptic stimulation without any postsynaptic depolarization (−4.5%, *n* = 1). The average of all non-overlapping burst cases is shown in Figure 2D, representing a statistically significant average decrease of −5.9 ± 1.4% in synaptic efficacy (*n* = 13, *p* < 0.01). Together, these observations demonstrate that retinal wave activity can evoke either homosynaptic potentiation or depression depending on burst latency (Figure 2E), and thus provides a mechanism for competition between inputs [31].

### Burst-Time–Dependent Plasticity over Second-Long Time Scales

**Figure 3:**
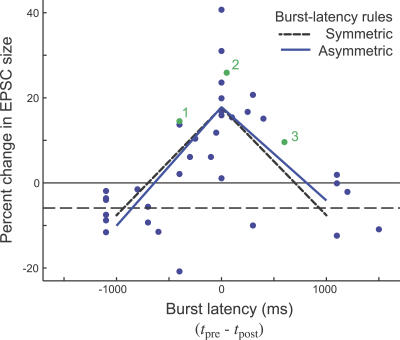
Burst-Time–Dependent Learning Rule at the Retinogeniculate Synapse Percent change in synaptic efficacy evoked by pairings at different latencies between pre- and postsynaptic bursts. The average change for non-overlapping bursts (Figure 2E) is shown as a dashed line. The best symmetric (solid) or asymmetric (dotted) burst-based rules are also shown. The three numbered examples are considered in detail in Figure 4. *t*_post_, time of the postsynaptic burst; *t*_pre_, time of the presynaptic burst.

Burst latencies between zero (fully overlapping) and ±1,000 ms (non-overlapping) evoked intermediate levels of plasticity. A summary of 39 experiments in which changes in synaptic efficacy could be reliably gauged is shown in Figure 3, and the results of an additional six experiments using 2-s pre- and postsynaptic bursts, which demonstrate consistent results, are shown in Figure S1. This burst-time–dependent learning rule is a concrete instance of Hebbian plasticity, since synaptic efficacy increases for “cells that fire together,” whereas synapses between neurons that are “out of sync” are weakened [22]. In fact, the tent-like shape of this learning rule demonstrates that—under the conditions studied—the total change in synaptic strength is related to the overlap between pre- and postsynaptic bursts, adjusted such that non-overlapping latencies result in synaptic weakening. Assuming that the overall burst latency, without regard to burst order, is linearly proportional to the observed changes in synaptic efficacy leads to the “symmetric” learning rule (dashed line) shown in Figure 3. To investigate whether the order of pre- versus postsynaptic bursting affected the amount of plasticity, we allowed a different linear relationship for positive versus negative latencies (solid line). However, the resulting “asymmetric” rule was not significantly different; thus it appears that burst order plays little role in determining synaptic changes.

**Figure 4:**
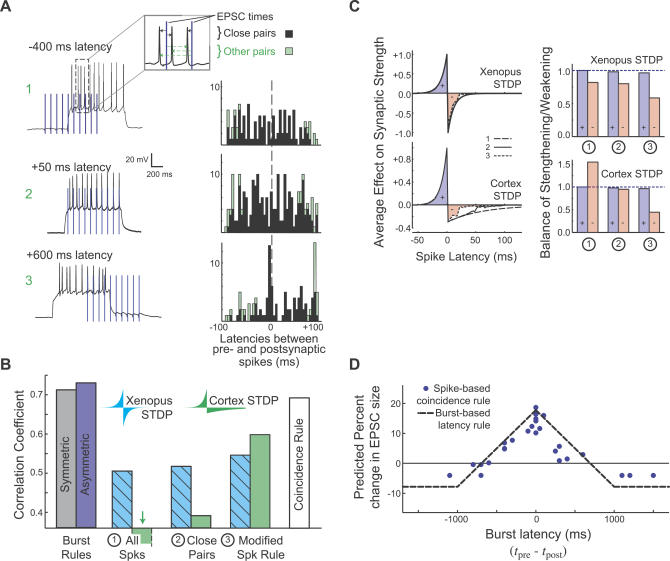
Spike-Timing–Based Rules Must Be Modified to Account for the Observed Plasticity (A) Histograms of spike-time latencies (right) measured from the timing between EPSPs and spikes evoked during the three different burst-based stimulation protocols shown (left). Nearest-neighbor times are shown in black, with additional non–nearest-neighbor times in green. (B) CCs demonstrating the degree to which different learning rules predict the observed data of Figure 3. Spike-based rules from the *Xenopus* retinotectal system [32] (hashed cyan) and mammalian somatosensory cortex [33] (green)—are compared with the burst-latency–based rules (left columns). Removing consideration of spike pairs that result in weakening produces better predictions: from the naively applied STDP (#1), to only considering nearest-neighbor spikes (#2), to the modified STDP that takes multi-spike interactions into account as suggested by Sjöstrom et al. [34] (#3). Removing the temporal window for depression entirely—leaving a simple spike-based coincidence rule—yields the best predictions (right column). (C) The ability of spike-based rules to predict the data in (Figure 3) is related to the degree to which depressing latencies are ignored. Left: the average *Xenopus* (top) and cortex (bottom) learning rule as more spike latencies are selectively ignored. Right: the resulting balance between strengthening (blue +) and weakening (red −) changes with successive modifications to STDP: the best predictions correspond to rules with the least amount of weakening. (D) The predictions of the spike-based learning rule derived from the total number of spike coincidences, compared with the burst-based rule that is just a function of burst latency (dashed line). *t*_post_, time of the postsynaptic burst; *t*_pre_, time of the presynaptic burst.

One of the most notable aspects of the observed burst-based learning rule is the second-long temporal window over which the magnitude of change in synaptic efficacy depends on the burst latency, and is in marked contrast to the much shorter temporal window (~10 ms) of spike-timing–dependent plasticity (STDP) [25]. The orders-of-magnitude difference in time scale between STDP and BTDP likely arises from the time scale of the bursts themselves, since a given burst pairing is associated with an ensemble of shorter latencies between pre- and postsynaptic spikes; three examples are shown in Figure 4A (corresponding to experiments numbered 1, 2, and 3 in Figure 3). It is therefore possible that the observed burst-based rule is the cumulative effect of a spike-based rule applied to the individual spike latencies that comprise a given burst pairing.

To discover whether a spike-based learning rule might underlie the observed BTDP, we evaluate the predictions of both spike-based and burst-based rules by calculating correlation coefficients (CC) between their predicted changes in efficacy and those observed in the 39 experiments of Figure 3 (see Materials and Methods). As a baseline, the burst-based learning rules of Figure 3—which assume the amount of synaptic change is linearly related to burst latency—yield CC = 0.71 for the symmetric rule and CC = 0.73 for the asymmetric rule (Figure 4B, first column).

To compare, we apply the spike-time–dependent learning rules measured in the *Xenopus* retinotectal system [32] and rat somatosensory cortex [33] to the measured spike-latency distributions (e.g., as shown in Figure 4A). First, we assume that each spike latency contributes independently to the total change in synaptic efficacy such that the all pairs of pre- and postsynaptic spikes that occur during each plasticity experiment (e.g., those comprising the spike-latency distributions shown in Figure 4A) can be used to generate a prediction of the total amount of synaptic change in each case. Doing so demonstrates that a naively applied STDP is a poor predictor of the observed plasticity, since the *Xenopus* rule has a correlation coefficient CC = 0.51, whereas the cortex rule is substantially worse and predicts synaptic strengthening when weakening was observed and vice versa, resulting in CC = −0.42 (Figure 4B, #1). These predictions improve, however, when only nearest neighbor (“close”) spike pairs are considered and the other pairs are ignored (Figure 4B, #2), especially for the cortex rule in which the long time window for depression usually encompasses many spike pairs.

In fact, previous studies of STDP in the context of more complicated neuronal activity [34,35] suggest phenomenological rules by which STDP can be modified to properly account for the observed synaptic plasticity. In particular, Sjöstrom et al. [34] suggest that, when individual spikes are associated with both positive and negative latency pairings, weakening is suppressed by strengthening. Applying this modified spike-based rule further improves the predictive power of both the *Xenopus* and cortex rules (Figure 4B, #3), though still not to the level of the burst-based rule. In particular, it leads to improvement by favoring strengthening over weakening for coincident bursts, which results in a net strengthening even though they have roughly equal numbers of positive (depressing) and negative (potentiating) spike pairs (e.g., see Figure 4A, middle). In this way, by ignoring the temporal window for depression, STDP predictions become increasingly consistent with the changes in synaptic efficacy observed in the context of the burst-based activity associated with retinal waves.

This is consistent with the idea that, in the context of the burst-based activity present in the developing visual system, the effective learning rule predicted by STDP itself changes its form. We demonstrate this by plotting the average spike-based learning rule as a function of spike latency (Figure 4C). First, in the naive application of STDP, every spike pair has its full effect, resulting in the normal STDP rules (Figure 4C, left, dashed lines). However, when only nearest neighbor spike pairs are considered, it becomes increasingly likely that longer-latency pairs will be ignored, and as a result, the average change in synaptic efficacy at a given latency is reduced. Because the bursts used in these experiments involve spike rates around 10 Hz (corresponding to roughly 100 ms in between each spike), the *Xenopus* rule is relatively unaffected by limiting the consideration to nearest neighbor spike pairs (Figure 4C, top, solid line), whereas longer positive latencies are attenuated for the cortex rule (Figure 4C, bottom, solid line). The Sjöstrom rule has a much great impact because it ignores positive-latency pairs that share a spike with negative-latency pairs, and results in a further attenuation of the window for synaptic weakening (Figure 4C, dotted lines). Overall, This leads to a significant decrease in amount of synaptic weakening predicted by STDP in the context of natural retinal wave activity (Figure 4C, right), especially for the cortex rule (Figure 4C, bottom) due to its long window of synaptic depression.

Notably, the ability of a spike-based rule to predict the observed plasticity seems to be inversely related to the amount of synaptic weakening predicted by the rule. As a result, we evaluated a simple spike-based coincidence rule—with no temporal window for synaptic weakening—to compare with the previous STDP rules. If *N* is the total number of spike latencies within 50 ms, the best fit to data is given by Δ = 0.15*N* − 4.05 (%), and has nearly the same predictive power (CC = 0.68) as the burst-based rules of Figure 3. The close correspondence of the predictions of this spike-based coincidence rule (Figure 4D) with the burst-based coincident rule (dashed line) suggests how BTDP—based on time scales on the order of a second (arguably most relevant to the slow propagating activity in the retina)—may be comprised of spike-based rules that act on shorter time scales that may be more appropriate for synaptic function.

### The Cumulative Effect of Retinal Waves on Retinogeniculate Synapses

These considerations together show that synaptic plasticity of retinogeniculate synapses resulting from “natural” activity patterns are directly predictable from the total amount of coincidence between pre- and postsynaptic activity, whether considering either spikes or bursts. As a result, though a given LGN neuron may receive tens to hundreds of RGC inputs driven by the complex spatiotemporal properties of retinal waves [16], synaptic development appears to be governed by a simple and robust computational principle: synaptic strengthening and weakening is proportional to the amount of coincident activity between RGC and LGN neurons. Does such “Hebbian” plasticity operate over time in a way that will drive retinal-wave–dependent development of the system?

**Figure 5:**
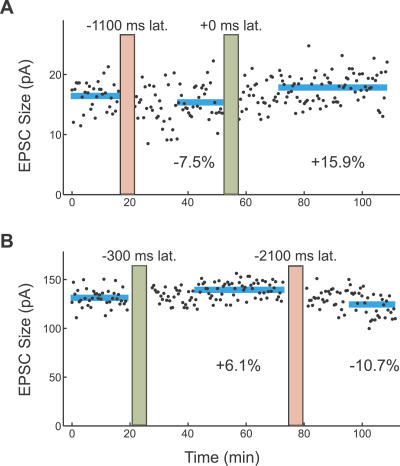
The Effects of Retinal Waves Are Cumulative over Time Two examples of experiments demonstrating the cumulative effect of two opposing stimulation protocols: (A) −1,100-ms latency (lat.) pairing followed by zero-latency pairing; and (B) −300-ms latency pairing followed by −2,100-ms latency pairing. Horizontal blue lines represent the mean EPSC size, and vertical green and red boxes demonstrate the duration of the stimulation protocol.

Activity-dependent retinogeniculate refinement occurs over several weeks in most species, meaning that the gradual strengthening of some synapses and elimination of most others is a cumulative effect of many thousands of individual retinal waves. Figure 5 provides evidence that the effects of retinal waves are indeed cumulative. These two examples show sequential induction of opposing plasticity with stimulation protocols separated by an hour: in the first example, a long-lasting weakening is followed by strengthening (Figure 5A); the reverse occurs in the second example (Figure 5B). These examples also provide another demonstration that the particular burst latency—rather than the mere presence of bursts—is responsible for determining the sign of plasticity.

**Figure 6:**
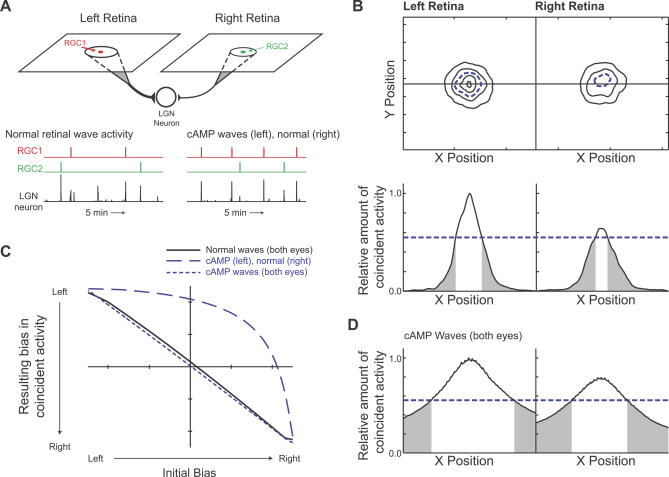
Observed “Hebbian” Plasticity Leads to Robust Retinogeniculate Refinement over Many Retinal Waves (A) Model schematic of localized areas in two retinas providing input to a single LGN neuron. Below: the LGN neuron activity is the sum of RGC activity over its inputs; a RGC from each eye and the LGN activity is shown for 5 min during a simulation with normal retinal waves (left) and a simulation in which the left eye has activity simulating raised cAMP levels, which results in increased wave size and frequency (right) [40]. (B) The amount of coincident activity between RGCs and the LGN neuron as a function of two-dimensional RGC position in the retina (top, contour plot) and a one-dimensional slice through the middle of each retina (bottom). The dashed horizontal line in each figure illustrates that for a given cutoff between strengthening and weakening, a larger number of RGCs in the left eye (compared with the right eye) will be strengthened in retinotopically appropriate positions. As a result, no matter where this cutoff is, more connections will be weakened (shaded areas) that originate from the right eye. (C) The results of simulations over a range of initial bias in connection strength between the eyes, demonstrating that the initial strength of connection biases competition in favor of the more strongly connected eye. Simulations were run using normal waves (solid line), as well as a condition simulating elevated cAMP levels in one eye (dashed line) or both eyes (dotted line). (D) The amount of activity overlap for simulations of elevated cAMP levels in both eyes (same format as in [B], bottom), showing that increasing wave size and frequency results in normal eye segregation as shown in (C), but less retinotopic refinement.

The cumulative effect of many retinal waves can be simulated across all the inputs to a given LGN neuron in a computer model of the retinogeniculate system (see schematic, Figure 6A). We model this system using the burst-based learning rule in conjunction with two simulated retinas composed of two separate sets of roughly 19,000 RGCs that independently generate retinal wave activity using a simulation that accurately reproduces their experimentally described spatiotemporal properties [16,36]. A subset of these RGCs from both retinas are initially connected to a single LGN neuron. Because both the activity of the RGCs and that of the LGN neuron [30] are comprised of bursts that last over seconds, and the resulting burst-based learning rule is correspondingly coarse, the particular details of how the LGN activity results from the input RGC activity do not qualitatively affect the simulation results. Furthermore, the cumulative changes in synaptic efficacy are predictable simply from the total amount of coincident activity between a given RGC and LGN neuron.

We simulate a short period of development (1,000 min) in order to demonstrate how retinal wave activity, combined with BTDP, drives eye segregation and refinement of retinotopy. By limiting this simulation to this short period, we can demonstrate clear developmental trends without needing assumptions about how the system evolves over longer times, which involves changes in retinal wave properties [14], functional changes in intra-LGN connectivity [37], and other aspects of longer-term development that are not experimentally constrained. Consider an LGN neuron that initially receives input from a localized set of RGCs in each retina (Figure 6A) with an initial bias towards the left eye (such that the right-eye connections are 20% weaker, but otherwise the same). The total amount of coincident activity between pre- and postsynaptic activity over the 1,000-min simulation is shown in Figure 6B as a contour plot over each retina (top) and also as a slice across the center of each retina (bottom). Due to the initially stronger connection to the left eye, retinal waves in the left eye drive more postsynaptic activity and, as a result, RGCs in the left eye will have, on average, more coincident activity. As a result, a dotted line representing a balance of strengthening and weakening (Figure 6B)—no matter its exact location—will cut through a relatively higher section of the right-eye curve, demonstrating that a disproportionate number of the right-eye connections will become weakened (shaded areas), and correspondingly more of the left-eye connections will become strengthened.

Furthermore, notice that the shaded areas in both eyes (Figure 6B, bottom) are furthest from the center of the retinal area that the LGN neuron is connected to. This occurs because RGCs at the center will most likely be involved in a retinal wave that evokes postsynaptic activity and thus have the most coincident activity. Thus, this simulation of a small segment of development demonstrates the longer-term trends of RGC refinement—driven by the observed BTDP—to become increasingly retinotopic and eye segregated. These trends arise simply because retinal wave activity correlates local regions of RGCs to become active together and thus cooperatively drive postsynaptic activity. Note that such development simply reinforces existing biases that are likely established through activity-independent mechanisms [38,39]: the tight correspondence between initial bias and amount of coincident activity is shown in Figure 6C (solid line).

The amount of area in the LGN occupied by RGC axons from each eye can also be influenced by differences in the activity between the eyes [20]. The retinal wave model can also simulate the aberrant retinal wave activity that is induced by raising intracellular cyclic adenosine monophosphate (cAMP) levels through the application of adenosine [36], which generates waves that are much more frequent and larger. We first simulated this aberrant activity in the left eye while keeping activity in the right eye normal, meaning that a given RGC in the left eye was involved in a retinal wave 2–3 times a minute (instead of once every 2–7 min), and more LGN activity is evoked during a given left-eye wave because of its larger size. This results in a larger amount of coincident activity in the left eye for a wide range of bias in the initial connection strength (Figure 6C, dashed line). These simulations thus reproduce the experimentally observed increase in territory of the more active eye [40]. Furthermore, when raised cAMP levels are simulated in both eyes, the amount of overlap again balances out (Figure 6C, dotted line), resulting in normal eye segregation as experimentally observed [20]. However, due to the large size of the waves in the cAMP condition, retinotopic refinement is much more coarse (Figure 6D), and as a result, may result in deficiencies in retinotopy.

Thus, this simple model—based on the observed synaptic learning rule in the LGN—illustrates both the robustness of eye segregation and retinotopic refinement driven by retinal waves, and also provides a larger framework to understand the many recent experiments manipulating retinal wave activity that have different outcomes for patterns of retinogeniculate connectivity.

## Discussion

Detailed studies of both retinogeniculate refinement and the retinal activity that drives it provide a unique opportunity to relate rules governing synaptic plasticity to their role in guiding activity-dependent development. Guided by this knowledge, we reproduced the relevant aspects of the population activity in an in vitro preparation of the LGN and OT. We discovered a novel learning rule based on the relative timing between bursts of action potentials in the presynaptic population of RGCs and the postsynaptic target LGN neurons, such that short latencies cause potentiation and longer ones cause depression. The changes in synaptic efficacy evoked by these “realistic” stimulation patterns have a gradual onset (Figure 2) and much smaller magnitude than a majority of synaptic plasticity observed with other stimulation protocols such as tetanus [24], and thus their effects are likely gradual and cumulative over the course of development (Figure 5).

One of the notable features of this observed BTDP is its seconds-long temporal window, which likely arises from the burst-based nature of the activity in the visual system at this stage of development [13,30]. Due to the compound aspect of this activity, we investigated whether it might arise from a shorter–time-scale rule such as STDP [25,32,33]. Though naively applied STDP cannot explain the observed plasticity, we found that modifications to STDP that account for multiple-spike interaction [34,35] result in much better predictions of the observed burst-based plasticity. Furthermore, the bursts present in retinal waves involve a much higher degree of multiple-spike interaction than has been studied [34,35], and in this sense, it is likely that the simple spike-based coincidence rule that best explains the observed plasticity (Figure 4C) may be an extreme form of modified STDP. However, since the form of STDP induced by isolated spikes is not known in this system, whether or not a spike-based coincidence rule is a modified form of a more traditional STDP rule (such as those measured in other systems, e.g., Figure 4) is a matter for future investigation.

Of course, the two examples of STDP considered in Figure 4 were observed in systems in which the action of single spikes is much more relevant: the “cortex STDP rule” is measured in the somatosensory cortex where single isolated spikes drive layer II/III neurons [41], and the *Xenopus* retinotectal STDP [32] is present at a time when vision, rather than retinal waves, drives RGC activity. Likewise, evidence suggests that the mammalian visual cortex is governed by STDP at later ages when activity is driven by vision [42], which results in less temporally and spatially correlated patterning of neuronal activity. In this way, the observations presented in this paper support how the relevant form of synaptic learning rules depends on the natural activity patterns that exist in a particular system.

The burst-based learning rule that we observe also resembles other forms of previously observed synaptic plasticity, and is strikingly similar to pairing protocols that induce LTP in other systems [43], because large synaptic currents that drive LGN neurons during retinal waves induce seconds-long depolarization [30], and we here show that the observed burst-based learning rule is well predicted by the number of presynaptic spikes paired with this depolarization (Figure 4C). In fact, tetanus-based plasticity also essentially amounts to depolarization paired with presynaptic stimulation (Figure 1C), though the magnitude and frequency of this stimulation makes it unclear how it can apply to natural activity patterns.

The ability to measure a learning rule that can be applied directly to known natural activity in the retinogeniculate system provides a framework for understanding a number of innovative recent experiments that have examined the link between retinal wave activity and patterning of RGC connections by disrupting natural retinal waves either pharmacologically or genetically [19–21,26,27]. Our observation that both homosynaptic strengthening and weakening are induced by natural activity patterns provides a mechanism for competition [31] that is necessary to understand the results of Stellwagen and Shatz [20] (Figure 6). In the meantime, there has been disagreement in the interpretation of experiments in which manipulations that disrupt retinal wave activity either prevented [11,21] or failed to prevent [19,21] eye segregation. Torborg et al. [21] concluded that high-frequency synchronized bursting between neighboring RGCs is necessary to drive eye segregation, whereas asynchronous spiking does not disrupt eye segregation. These results are entirely consistent with BTDP: high-frequency bursting among neighboring RGCs would elicit postsynaptic activity in connected LGN neurons and strengthen existing connection biases, whereas asynchronous spiking would not evoke postsynaptic activity. However, our model suggests that the crucial factor determining whether RGC axons from each eye segregate in the LGN is not activity between pairs, but the summed activity over local regions of the retina that would drive LGN activity (which should be observable in the retina with multi-electrode or imaging). In this context, disrupted eye segregation might only be observed in conditions where RGC activity cannot consistently elicit postsynaptic spiking.

The observed burst-time–dependent plasticity provides a simple computational principle for organizing an immature system driven by spontaneous activity; in fact a burst-based learning rule would be “safer” at developing synapses, where vesicle release is slow and more uncertain [44]. Furthermore, the simplicity and robustness of this rule suggests that it may exist at other developing visual synapses driven by retinal waves that have been shown to require retinal wave activity to drive refinement, including the visual cortex [7,8] and superior colliculus [9].

Such a Hebbian learning rule has been predicted at this synapse because the first observations of retinal waves [13,14,22], and several modeling studies of the retinogeniculate system [45–47], have shown that a Hebbian rule could use retinal wave activity to instruct refinement. In fact, a learning rule of this nature makes effective use of the information provided by the detailed spatiotemporal properties of the retinal waves [1,17]. Such a match between the information conveyed by retinal waves and the properties of the observed plasticity suggests a system-level organizational strategy in which the properties of retinal waves and activity-dependent plasticity are tuned to each other in order to drive and maintain activity-dependent development robustly.

## Materials and Methods

### Slice preparation.

Brain slices containing the LGN and OT were prepared from rat pups (P7–11). They were 350 μm thick, and cut in a tipped parasagittal plane [48]. The bathing solution (ACSF) contained (in mM): 130 NaCl, 3 KCl, 1.25 KH_2_PO_4_, 20 NaHCO_3_, 10 glucose, 1.3 MgSO_4_, 2.5 CaCl_2_ (pH 7.4, equilibrated with 95%O_2_–5% CO_2_). Slices were held in a recording chamber on a fixed stage microscope and superfused (3–5 ml/min) with ACSF at room temperature (~25 °C). Though no inhibitory postsynaptic currents (IPSCs) were observed during recording for ages under P12 despite using a low-chloride internal solution (see below), picrotoxin (100 mM) was added in a subset of experiments to the ACSF to block fast GABAergic transmission.

### Perforated patch recording.

Perforated patch current and voltage clamp recordings were made with electrodes pulled from borosilicate glass with a final resistance of 3–7 MΩ. The recording electrodes contained (in mM): 100 K-gluconate, 4 NaCl, 20 KCl, 0.2 CaCl_2_, 10 HEPES (free acid), 1.1 ethylene glycol-bis (b-aminoethyl ether)-N,N,N′,N′-tetraacetic acid (EGTA), 2 Mg-ATP, 1 MgCl_2_ and 5 glutathione (pH 7.2), 50 mg/ml amphotericin, and (for some experiments) Lucifer Yellow (K-salt, ~1 mg/ml; Molecular Probes, Eugene, Oregon, United States). Recordings were made with either an Axopatch 200B or 700A amplifier (Axon Instruments/Molecular Devices, Sunnyvale, California, United States), digitized using a 16-bit A/D converter at 5–10 kHz and filtered at 2 kHz. All voltages were adjusted for an estimated electrode-bath junction potential of −12 mV by offline subtraction. Bridge correction was performed offline. Digitized data were analyzed by MATLAB (Mathworks, Natick, Massachusetts, United States).

Recordings were made from LGN neurons identified using differential interference contrast (DIC) visualization. Once a seal was obtained (>1 GΩ), access resistance was monitored as it decreased (due to the amphotericin) and stabilized from 30–100 MΩ, at which point the synaptic plasticity experiments (described below) were commenced. Since a large problem in measuring plasticity was an accidental rupture of the patch during the experiment, several methods were used to control for this. Initially, Lucifer Yellow was included in the internal solution and was imaged using fluorescence microscopy every 30 s to verify that the membrane patch did not rupture (which would result in the neuron filling). Recent experiments used an electrode brace attached directly to the headstage that increased electrode stability and removed most fluctuations in serial resistance, making a patch rupture identifiable from jumps in the access resistance alone.

### Synaptic plasticity experiments.

A bipolar stimulating electrode was placed into the OT. Stimulus current was turned down until it did not elicit input to the LGN neuron recorded using perforated patch, and then gradually turned up to until a stable EPSC was seen (7–140 pA). This “minimal stimulation” arguably stimulates one or few synapses, but in many cases the amount of input varied continuously with stimulus current, in which case the current was adjusted to where the EPSC size was relatively stable. In every case, the EPSC represented a small fraction of the total input to the LGN neuron, because turning up the OT stimulus current could evoke significantly larger currents, which could be a nanoamp or greater.

The EPSC amplitude was monitored in an LGN neuron in voltage clamp every 30 s until a stable baseline was achieved. The recording mode was then switched to current clamp, and 1-s current steps of increasing amplitude were delivered until the neuron was driven to fire 10–20 spikes in the second interval. Then, 10 Hz OT stimulation was paired with this current injection at a prescribed latency, and this was repeated ten times at 40-sec intervals (Figure 1). The recording mode was then switched back into voltage clamp, and the resulting EPSC amplitude was monitored as it had been during the baseline recording. Serial resistance was constantly monitored over the experiment, and trials in which there was significant change (denoting patch rupture) or in which smaller changes in *R*_S_ were correlated with changes in EPSC size were discarded.

### Spike-time-based learning rules.

A given stimulation protocol evokes excitatory postsynaptic potentials (EPSPs) at precisely determined times {*t*_E_} determined by the times of OT stimulation adjusted by the synaptic latency determined during baseline recording. In the meantime, the timing of evoked LGN spikes {*t*_spk_} are directly observable from the current clamp recordings during the stimulation protocol. Together, these timings were used to form histograms of the latencies {*L* = *t*_E_ − *t*_spk_} of three different classes of spike pairs: (1) comparisons of timings between all EPSPs and spikes; (2) comparison between nearest-neighbor spikes only; and (3) comparison between selected pairs of nearest neighbor spikes as suggested by Sjöstrom et al. [34], which ignore pairs with positive latencies when either of the spikes in the pair was also paired with other spikes with a negative latency.

These spike latency histograms—one set for each experiment—were then used to derive spike-time–dependent learning rule predictions based on a wide class of STDP-type rules. In particular, for a particular set of latencies {*L*_i_}, the *predicted* change in synaptic efficacy Δ*^P^* for a general spike-time-based rule is given by:

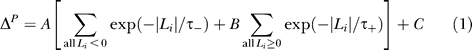
Note that the constants *A* and *C* adjust the overall scaling and offset of the rule such that the resulting plasticity Δ^P^ can be matched to the experimental data of Figure 3. *A* and *C* are chosen to obtain the best fits for each learning rule, and do not affect the CC. Note that *C* can be negative, implying that that non-overlapping bursts—which may not be associated with any spike latencies in the relevant window—would result in synaptic weakening given by *C*. In our results, we report results from rules measured in the retinotectal system (*B* = −1, τ_−_ = τ_+_ = 10 ms [32]); and somatosensory cortex (*B* = −0.3, τ_−_ = 10 ms, τ_+_ = 60 ms [33]). Local variations of the parameters of these rules were also investigated and did not yield significantly better fits. We also applied the rules for positive values for *B* in each case, as well as a rule that uses only the set of latencies *N*_L_ within 50 ms. The best rule in this case was Δ = 0.145*N*_L_ − 4.0472 (%).

We evaluated the fits of the learning rules using two methods: CC and mean-squared error (MSE). The CC compared the observed plasticity data for all 39 experiments (*j* = 1 to 39) {Δ^0^ *_j_*} to the predicted plasticity {Δ^P^ *_j_*} using CC = ∑*_j_* [Δ^0^ *_j_* − mean(Δ^0^)] [Δ^P^ *_j_* − mean(Δ^P^)]/[std(Δ^0^)std(Δ^P^)]. A CC of 1 means that the data are perfectly predicted by the learning rule, and zero means that it is completely uncorrelated. The MSE is given by MSE = ∑*_j_* [Δ^0^ *_j_* − Δ^P^ *_j_*]^2^. Because of the correspondence of the results of these two methods and the lack of dependence of the CC on the parameters *A* and *C,* we reported only CC results here.

### Burst-time–based learning rules.

We also used the CC and MSE to determine the best burst-based learning rule. The best symmetric rule as a function of burst latency *L*_B_ is given by Δ = 18.2 − 25.8 *L*_B_ (for |*L*_B_| < 1), and Δ = −7.6 for |*L*_B_| ≥ 1. The best asymmetric rule used 18.3% for zero intercept and −28.9 and −21.0 (%/sec) for the left and right slopes, respectively.

### Retinogeniculate model.

A model of two retinas and a single LGN neuron were simulated as described in the Results (Figure 6A). To generate retinal waves in each retina, we implemented the retinal wave model of Butts et al. [36], which ran for 1,000 simulated minutes using two different random number seeds to simulate uncorrelated retinal wave activity in each eye. The weight matrices *w*_L,*i*_ and *w*_R,*i*_ that determine the initial strength of connection between the LGN neuron and each RGC in both eyes were given by two-dimensional Gaussian distributions centered at the center of each retina with a standard deviation of ten times the RGC cell spacing, and their overall magnitude was multiplicatively scaled to establish an initial bias. LGN activity at every time step *R*(*t*) was given by:

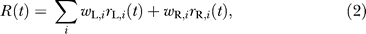
where *r*_L,*i*_(*t*) and *r*_R,*i*_(*t*) are either 0 or 1 depending on whether the given RGC is bursting or not at time *t*. Note that particular physiological details of the LGN neuron only affect its activity over shorter time scales, and thus this general model for activity is sufficiently representative of a large range of conditions for the purposes of this simulation. The amount of coincident activity between a given RGC and the LGN neuron is then given by O_L,R_ = ∑*_t_ R*(*t*) *r*_L,R,*i*_(*t*), and is plotted in Figure 4 for various simulations. The “resulting bias in coincident activity” (Figure 6C) is on a scale of zero (right eye) to one (left eye) and is given by O_L_/(O_L_+O_R_). Note that the reported results occur robustly for a large range of initial connectivities, and manipulating the few assumptions of this model do not qualitatively affect our results.

The manipulated waves simulating conditions of increasing cAMP [40] represented a different parameter regime of the same retinal wave model [36], and specifically, we used the spontaneous rate *p* = 0.002 and the threshold θ = 2.2 instead of the normal *p* = 0.030 and θ = 3.5.

All simulation code is available at the Web site http://rd.plos.org/10.1371_journal.pbio.0050061_01.

## Supporting Information

Figure S1 The Effect of Longer Burst Duration Burst duration is very variable from wave to wave, and does not contain any developmentally relevant information. Here, we show the results of six pilot experiments using 2-s bursts in place of 1-s bursts (black x inside circles) yield qualitatively similar results compared to what might be expected from the 1-s burst experiments (blue). (The standard deviation of the 1-s bursts is shown as vertical blue lines.) Though these experiments are highly variable—like the one-second burst experiments—overlapping bursts result in synaptic strengthening, and non-overlapping bursts result in a mild weakening. The variability of the data prevents statistically significant comparisons, but it is likely that the changes in synaptic efficacy are larger in the case of 2-s bursts.
(232 KB PDF)

## Abbreviations

BTDP: burst-time–dependent plasticity
cAMP: cyclic adenosine monophosphate
CC: correlation coefficient
EPSC: excitatory postsynaptic current
EPSP: excitatory postsynaptic potential
LGN: lateral geniculate nucleus
OT: optic tract
RGC: retinal ganglion cell
R_S_: serial resistance
STDP: spike-time–dependent plasticity
