# Supplementary material for: A Burst-Based “Hebbian” Learning Rule at Retinogeniculate Synapses Links Retinal Waves to Activity-Dependent Refinement
Source: PLoS Biol. 2007 Mar 6;5(3):e61. doi: 10.1371/journal.pbio.0050061 (PMC1808114; doi:10.1371/journal.pbio.0050061)
Supplement: Supplementary file 1 [file pbio.0050061.sg001.pdf]

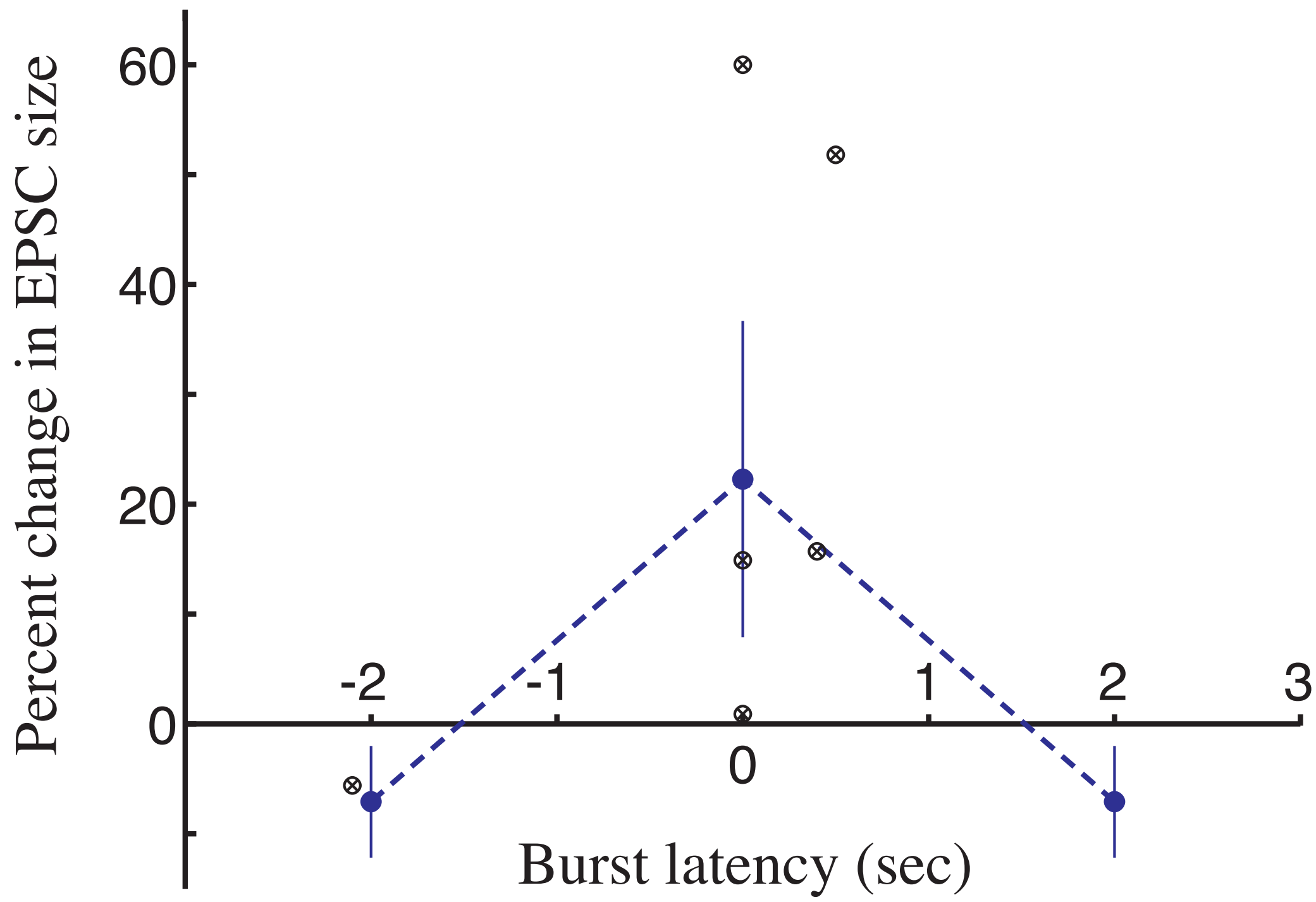

Percent change in EPSC size
60
40
20
0
-2
-1
0
1
2
3
Burst latency (sec)
